# A Grafting Strategy for the Design of Improved G-Quadruplex Aptamers and High-Activity DNAzymes

**DOI:** 10.1371/journal.pone.0005126

**Published:** 2009-04-09

**Authors:** Tao Li, Erkang Wang, Shaojun Dong

**Affiliations:** 1 State Key Laboratory of Electroanalytical Chemistry, Changchun Institute of Applied Chemistry, Chinese Academy of Sciences, Changchun, Jilin, China; 2 Graduate School of the Chinese Academy of Sciences, Beijing, China; University of Helsinki, Finland

## Abstract

Nucleic acid aptamers are generally obtained by *in vitro* selection. Some have G-rich consensus sequences with ability to fold into the four-stranded structures known as G-quadruplexes. A few G-quadruplex aptamers have proven to bind hemin to form a new class of DNAzyme with the peroxidase-like activity, which can be significantly promoted by appending an appropriate base-pairing duplex onto the G-quadruplex structures of aptamers. Knowing the structural role of base pairing, here we introduce a novel grafting strategy for the design of improved G-quadruplex aptamers and high-activity DNAzymes. To demonstrate this strategy, three existing G-quadruplex aptamers are chosen as the first generation. A base-pairing DNA duplex is grafted onto the G-quadruplex motif of the first generation aptamers. Consequently, three new aptamers with the quadruplex/duplex DNA structures are produced as the second generation. The hemin-binding affinities and DNAzyme functions of the second generation aptamers are characterized and compared with the first generation. The results indicate three G-quadruplex aptamers obtained by the grafting strategy have more excellent properties than the corresponding original aptamers. Our findings suggest that, if the structures and functions of existing aptamers are thoroughly known, the grafting strategy can be facilely utilized to improve the aptamer properties and thereby producing better next-generation aptamers. This provides a simple but effective approach to the design of nucleic acid aptamers and DNAzymes.

## Introduction

Aptamers are nucleic acids (DNA or RNA) that bind any given molecular target with high affinity and specificity. They are generally selected from the pools (∼10^15^) of random sequences through an *in vitro* evolution process termed SELEX (systematic evolution of ligands by exponential enrichment) [1], [2]. In comparison with protein antibodies, nucleic acid aptamers are more easily obtained, modified and manipulated. The specific targets recognized by aptamers cover metal ions [3], [4], small molecules [5]–[7], proteins [8]–[12], and even whole cells [13], [14] or viruses [15], [16]. These unique properties endow aptamers with great application potential in clinical therapeutics [17], biological researches [18], molecular recognition [19], bioanalysis and sensing [20], [21]. This is attracting more and more efforts directed to the development of new functional aptamers, such as catalytic aptazymes consisting of an aptamer domain and a ribozyme module [22]. Because of their enzyme functions, aptazymes are widely applied to the detection of various targets that specifically bind to the aptamer domain and, in general, cause a conformational change [23], [24]. Interestingly, a few specific DNAs are able to serve as both aptamers and ribozymes. They are originally selected by SELEX as the aptamers for anionic porphyrins [7]. Then, they are proven able to catalyze the metallation of mesoporphyrin IX by copper and zinc ions [25]. These specific DNA aptamers have G-rich sequences with ability to form four-stranded structures known as G-quadruplexes that are crucial for ligand binding and biocatalysis. In particular, such G-quadruplex aptamers have another important function, i.e., the peroxidase-like DNAzyme function, which is the emphasis of this study.

DNAzymes (also called DNA enzymes, deoxyribozymes or catalytic DNAs) are artificial enzymes with great promise in biochemical and biotechnological applications [26]–[28]. An important kind of DNAzymes combining G-quadruplex DNA aptamers with hemin reveals the peroxidase-like activity [29]–[31]. They are able to catalyze the H_2_O_2_-mediated oxidation of 2,2′-azino-bis(3-ethylbenzothiazoline-6-sulfonic acid)diammonium salt (ABTS) [29], [30] or luminol [32], [33]. This feature enables the utilization of G-quadruplex-based DNAzymes for colorimetric or chemiluminescence detection of various analytes [32]–[39]. However, only a few G-quadruplex aptamers have been reported to exhibit excellent DNAzyme functions [29], [33], [34]. So it is interesting and significant to improve the intrinsic properties of G-quadruplex DNA aptamers, thereby producing some better ones.

In fact, some efforts have been made to improve the ligand-binding affinities of G-quadruplex aptamers [9], [40]. These studies indicate appropriate base-pairing duplexes that flank the G-quadruplex structures of thrombin-binding aptamers contribute to high-affinity binding. Base pairing has also proven a magic hand that controls the folding of G-quadruplex DNAs and regulates their intrinsic properties [41]. An appropriate base-pairing duplex flanking the G-quadruplex is found to confer higher hemin-binding affinity and more excellent DNAzyme function. This means that base pairing contributes to not only high-affinity binding but also DNAzyme formation. The promotion effect of base pairing on the G-quadruplex-based DNAzyme is also observed in another of our previous works [35]. These findings reveal that the base-pairing duplex plays an important role in the G-quadruplex structures.

Knowing the structural role of base pairing, herein, we introduce a novel grafting strategy for the design of improved G-quadruplex aptamers and peroxidase-like DNAzymes. It can be described as an artificial procedure in which an appropriate base-pairing duplex of one existing aptamer is grafted onto another one to produce the next generation. This strategy is demonstrated by the design of three new DNA aptamers with the quadruplex/duplex structures. The experimental observations show the next generation aptamers all exhibit higher binding affinity and better DNAzyme function than the corresponding original aptamers.

## Results and Discussion

**Figure 1:**
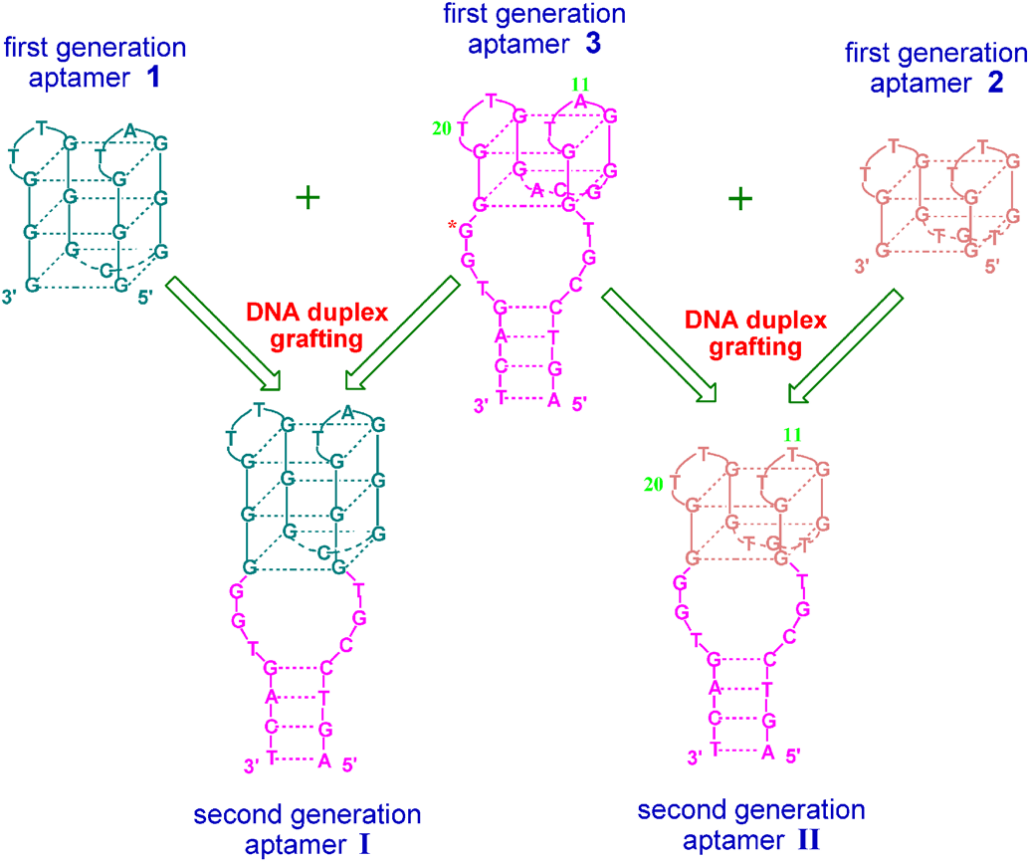
Schematic of the grafting strategy for aptamer design. The DNA duplex containing Watson-Crick base pairs of the first generation aptamer 3 is grafted onto the G-quadruplex structures of 1 and 2 to produce two new quadruplex/duplex DNA structures I and II as the second generation aptamers.

Figure 1 depicts how to design new G-quadruplex aptamers through the grafting strategy. Three existing G-quadruplex aptamers [8], [9], [33] are chosen as the first generation aptamers **1**, **2**, **3**. The aptamer **3** has a DNA duplex consisting of four Watson-Crick base pairs and two 3-nucleotide spacers. This DNA duplex can be grafted onto the G-quadruplex structures of **1** and **2**, thus two new quadruplex/duplex DNA structures are produced as the second generation aptamers **I** and **II**.

**Figure 2:**
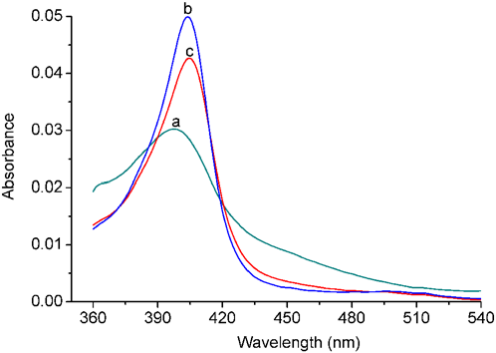
UV−Vis absorption spectra for analyzing the interactions between hemin and the aptamers 1 and I in the HEPES buffer. (a) 0.5 µm hemin; (b) incubation a with 0.5 µm of the aptamer 1; (c) incubation a with 0.5 µm of the aptamer I.

The second generation aptamer **I** has a G-quadruplex core the same as that of the aptamer **1** [33], which is responsible for hemin binding. As we know, the binding of DNA G-quadruplexes to hemin is able to cause an obvious hyperchromicity of the Soret band of hemin [29], [30], [42], [43]. This characteristic is here utilized to investigate the hemin−aptamer interactions. Figure 2 shows the UV−Vis absorption spectra of hemin before and after incubation with the aptamers **1** or **I**. The uncomplexed hemin has a Soret absorption band centered at 397 nm (curve a). Upon incubation with the aptamer **1** or **I**, a noticeable hyperchromicity is observed in the hemin Soret band (curves b, c). The absorption center shifts to 404 or 405 nm, accompanied by an observable increase in the absorption intensity. This indicates the binding of the G-quadruplex aptamer **1** or **I** to hemin.

The hemin-binding affinities of aptamers **1** and **I** are determined as described previously [41]. The results reveal that, in our experimental conditions, the aptamer **I** binds hemin with a submicromolar affinity (*K*_d_ = 194±10 nm), which is higher than that of the aptamer **1** (*K*_d_ = 258±7 nm). That is, the DNA duplex flanking the G-quadruplex contributes to high-affinity binding, consistent with previous observations [9], [41].

**Figure 3:**
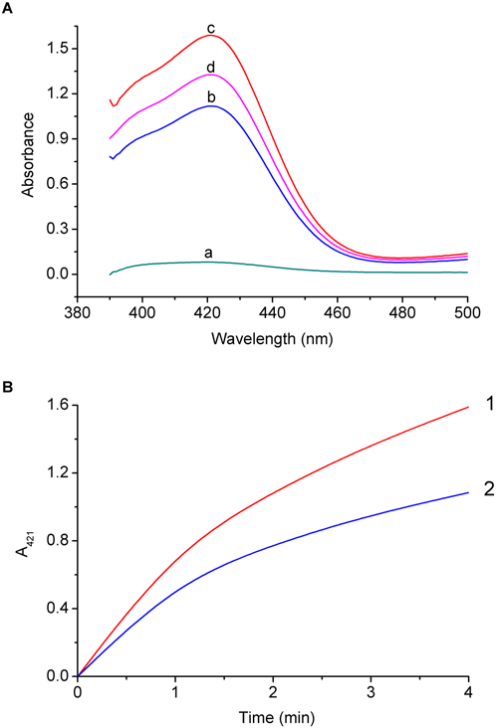
Comparison between the DNAzyme functions of the aptamers 1 and I. (A) UV−Vis absorption spectra (after 4 min) for analyzing 0.5 µM catalysts with the ABTS−H_2_O_2_ colorimetry in the detection buffer: a) hemin, b) hemin plus the aptamer 1, c) hemin plus the aptamer I, d) hemin plus a control DNA obtained by replacing the G residues of two spacers in the aptamer I with T. (B) Reaction kinetics of the H_2_O_2_-mediated ABTS oxidation catalyzed by: 1) the hemin−I DNAzyme, 2) the hemin−1 DNAzyme.

We then draw a comparison between the DNAzyme functions of the aptamers **1** and **I** in the ABTS−H_2_O_2_ reaction system (Figure 3). The oxidation of ABTS by H_2_O_2_ can produce the cationic free radical ABTS^+^, which has a maximal absorption at about 421 nm. Thus, the absorbance at this wavelength (A_421_) can be utilized to quantitatively analyze the oxidation product and evaluate the DNAzyme functions of different G-quadruplex aptamers. Figure 3A shows the uncomplexed hemin has a very low catalytic activity towards the H_2_O_2_-mediated ABTS oxidation (curve a), whereas the catalytic activity increases sharply upon incubation with the aptamer **1** or **I** (curves b, c). This indicates the formation of hemin−G-quadruplex DNAzyme. In general, the DNAzyme functions of G-quadruplex aptamers are quantitatively evaluated by the initial catalytic rates (denoted by ν) [29], [30], [41]. The ν values of **1** and **I** can be obtained from the corresponding kinetic curves of enzymatic reactions (Figure 3B). It is found that the aptamer **I** has a higher DNAzyme function (ν = 20.8 µm/min) than that of **1** (ν = 15.3 µm/min). That is, the DNAzyme function is improved by about 36% *via* grafting the DNA duplex onto the G-quadruplex structure of **1**.

Further experiments reveal this improvement in the DNAzyme function of the aptamer **I** is attributed to not only four Watson-Crick base pairs but also the two specific nucleic acid spacers, i.e., the residues of two spacers have influence on the DNAzyme activity. Figure 3A (curve d) shows that, when the G residues of two spacers are all replaced by T, the resulting quadruplex/duplex DNA structure exhibits a relatively low DNAzyme function as compared with the aptamer **I** whereas it is still higher than that of **1**. Kubik *et al.* have indicated the first G residue of the 3′-spacer (indicated by an asterisk in Figure 1) most possibly contributes to high affinity of the aptamer **3** [9]. Our observations also suggest that the G residues of two spacers of the aptamer **I** play an important role in hemin binding and DNAzyme formation.

**Figure 4:**
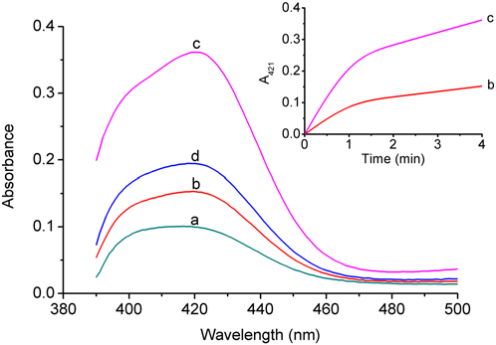
UV−Vis absorption spectra (after 4 min) for analyzing the DNAzyme functions of different G-quadruplex aptamers: a) 1 µm hemin, b) a plus 1 µm of the aptamer 2, c) a plus 1 µm of the aptamer II, d) a plus 1 µm of the aptamer 3. The inset shows the reaction kinetics corresponding to the aptamer 2 (b) and the aptamer II (c).

The second generation aptamer **II** contains the 15-nucleotide “core” sequence the same as that of the aptamer **2**, which is responsible for thrombin binding. Macaya *et al.* have confirmed that all antithrombotic oligonucleotides with a uniform G-quadruplex core will bind the same exosite of thrombin [40], so the aptamer **II** is thought to bind the electropositive fibrinogen recognition exosite of thrombin, as the aptamer **2** does [44]. In addition, the antithrombotic oligonucleotides with the quadruplex/duplex structures generally possess 4–10 times higher affinity than the G-quadruplex structures without duplexes [40]. This means the thrombin-binding affinity of the aptamer **II** is over 4-fold higher than that of the aptamer **2**, mainly owing to the negatively charged DNA duplex that enables the aptamer **II** to bind thrombin more strongly. Interestingly, the aptamer **II** is also able to bind hemin to form the G-quadruplex-based DNAzyme, and the introduced DNA duplex is found to promote the DNAzyme function of the aptamer **II**. Figure 4 depicts a comparison between the DNAzyme functions of **II** and **2** in the ABTS−H_2_O_2_ system. The inset shows the corresponding reaction kinetics, from which the ν values of the two aptamers **II** and **2** are obtained. It is observed that the aptamer **2** has a very low DNAzyme function (ν = 2.76 µm/min), which is about 2.5 times lower than that of the aptamer **II** (ν = 6.67 µm/min). That is, there is a ca. 150% improvement in the DNAzyme function after grafting the DNA duplex onto the G-quadruplex structure of **2**.

The aptamers **3** and **II** have very similar quadruplex/duplex DNA structures. However, the former exhibits a relatively low DNAzyme function as compared with the latter (Figure 4, curve d). This can be reasonably interpreted in terms of the potential interaction between loop residues of G-quadruplexes. A previous study [45] has indicated that, in the G-quadruplex structure of the aptamer **2**, a potential T−T base pair is formed between the two TT loops across the diagonal of the top G-tetrad. This additional base pair has a little contribution to the stability of the G-quadruplex structure. Accordingly, the interaction between two loop residues T_11_ and T_20_ may also occurs in the G-quadruplex structure of the aptamer **II**, whereas substitution of T_11_ to A_11_ in the aptamer **3** no longer allows this T−T base pair to form [9]. So the aptamer **II** has a more stable G-quadruplex core than **3**, which may account for the difference between the DNAzyme functions of two aptamers.

**Figure 5:**
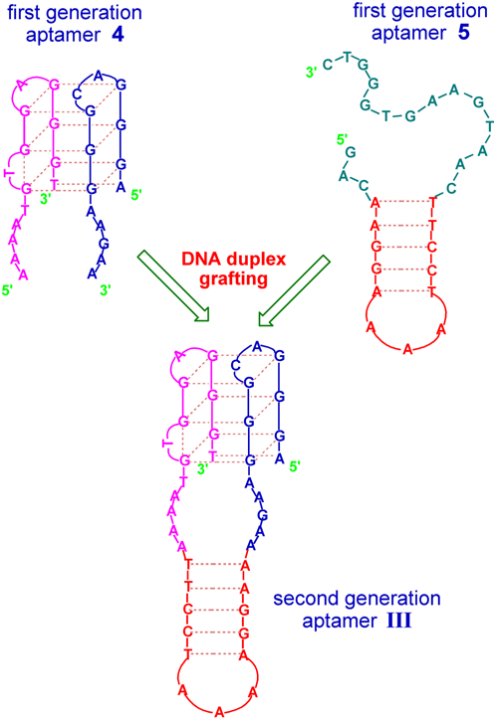
Design of the second generation aptamer III through the grafting strategy.

To further demonstrate the generality of the grafting strategy, other two first generation aptamers **4** and **5** are chosen to engineer another new aptamer **III** (see Figure 5). A 30-mer anti-cocaine aptamer **5** [46] contains a hairpin-like DNA duplex consisting of five Watson-Crick base pairs and a 3-nucleotide loop. This DNA duplex can be grafted onto a bimolecular G-quadruplex aptamer **4** [47], thus another DNA aptamer **III** with the quadruplex/duplex structure is produced as the second generation.

**Figure 6:**
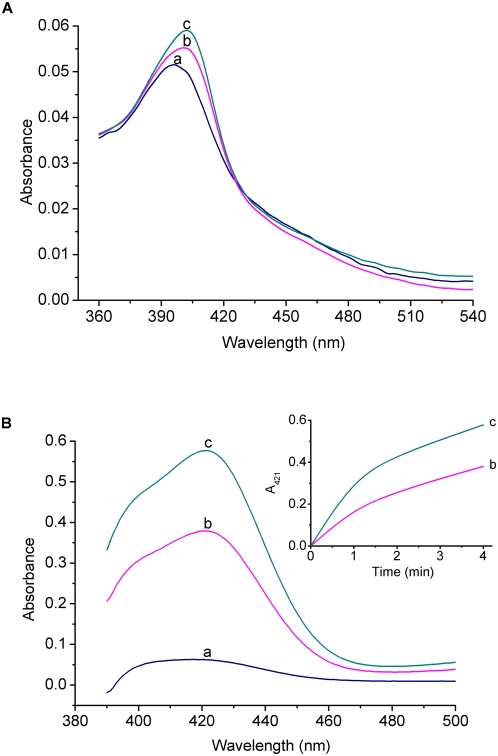
Comparison between the main properties of the aptamer 4 (b) and III (c), with the uncomplexed hemin (a) as a reference. (A) UV−Vis absorption spectra of the hemin−G-quadruplex complexes (1 µm) corresponding to the aptamer 4 and III in the HEPES buffer. (B) Spectroscopic analysis of the DNAzyme functions of the aptamers 4 and III (0.5 µm) with the ABTS−H_2_O_2_ colorimetry in the detection buffer. The inset shows the reaction kinetics corresponding to the aptamer 4 (b) and the aptamer III (c).

Similarly, UV−Vis absorption spectroscopy is here utilized to investigate the hemin-aptamer interactions, and the binding of G-quadruplex aptamers to hemin is indicated by a hyperchromicity of the hemin Soret band (Figure 6A). The aptamer **III** is found to exhibit an about two times higher affinity than the aptamer **4**. Furthermore, when investigated with the ABTS−H_2_O_2_ colorimetry (Figure 6B), the aptamer **III** also reveals a relatively high DNAzyme function (ν = 9.01 µm/min) as compare with the aptamer **4** (ν = 4.96 µm/min). According to these ν values obtained from the corresponding reaction kinetics (Figure 6B, inset), there is an about 82% improvement in the DNAzyme function after grafting the hairpin-like DNA duplex onto the G-quadruplex structure of the aptamer **4**. The control experiments reveal that, in the quadruplex/duplex structure of the aptamer **III**, the residues of two spacers linking the G-quadruplex motif and base-pairing duplex also have a noticeable influence on the DNAzyme function (data not shown). These observations further confirm the usefulness and generality of the grafting strategy for the design of improved G-quadruplex aptamers and high-activity DNAzymes.

In addition to the above-mentioned aptamers **1**, **2** and **4**, there are many G-rich DNA aptamers with ability to form the G-quadruplex structures under appropriate conditions. We hypothesize that the grafting strategy can be also utilized to improve the properties of these G-quadruplex aptamers, thereby producing more ones with better properties.

## Materials and Methods

### Oligonucleotides

The first generation aptamers (**1**, 5′ GGG TA GGG C GGG TT GGG 3′; **2**, 5′ GG TT GG TGT GG TT GG 3′; **3**, 5′ AGTC CGT GG TA GG GCA GG TT GG GGT GACT 3′; **4**, 5′ AGGG AC GGGA AGAA 3′ and 5′ AAAA TGTGG A GGGT 3′; **5**, 5′ GACA AGGA AAA TCCT TCA ATG AAG TGG GTC 3′), and the second generation aptamers (**I**, 5′ AGT CCG TGG GTA GGG CGG GTT GGG GGT GAC T 3′; **II**, 5′ AGT CCG TGG TTG GTG TGG TTG GGG TGA CT 3′; **III**, 5′ AGG GAC GGG AAG AAA AGG AAA ATC CTT AAA ATG TGG AGG GT 3′) were synthesized by Sangon Biotechnology Co., Ltd (Shanghai, China). These single-stranded oligonucleotides were dissolved in the TE buffer (10 mm Tris−HCl, 0.1 mm EDTA, pH 7.4), and quantified by using UV−Vis absorption spectroscopy with the following extinction coefficients (ε_260nm_, m ^−1^ cm^−1^): A = 15400, G = 11500, C = 7400, T = 8700.

### Preparation of hemin−G-quadruplex complexes

Before use, the DNA solutions were heated at 88°C for 10 min to dissociate any intermolecular interaction, and gradually cooled to room temperature. An equal volume of the 2× HEPES buffer (50 mm HEPES, 40 mm KCl, 400 mm NaCl, 0.1% Triton X-100, 2% DMSO, pH 7.4) was added to the DNA solutions, and allowed DNA sequences to properly fold for 40 min. Finally, an equivalent of hemin in the HEPES buffer (25 mm HEPES, 20 mm KCl, 200 mm NaCl, 0.05% Triton X-100, 1% DMSO, pH 7.4) was added into the above DNA solutions, and incubated for over 1 h to form hemin−G-quadruplex complexes.

### Spectroscopic analysis of DNAzymes

All colorimetric measurements were performed at room temperature in the detection solution (25 mm HEPES, 20 mm KCl, 200 mm NaCl, 0.05% Triton X-100, 1% DMSO, pH 8.0) by using UV−Vis absorption spectroscopy. Briefly, to 980 µL of 6 mm ABTS solution was added 10 µL of 60 mm H_2_O_2_. Then, 10 µL of DNAzyme was quickly added to this mixture. The absorption spectra of the reaction mixture were recorded every 1 min with a Cary 500 Scan UV−Vis−NIR Spectrophotometer (Varian, USA) in the wavelength range from 390 to 500 nm.

### Binding assays

We have developed a spectroscopic method for determining the affinities (*K*_d_) of hemin-binding aptamers in our previous work [41]. Briefly, 0.1 µm hemin was incubated with different concentrations of aptamers for 1 h in the aqueous buffer consisting of 5.45 mm ABTS, 25 mm HEPES (pH 8.0), 20 mm KCl, 200 mm NaCl, 0.05% Triton X-100, and 1% DMSO. Then, to 990 µL of the hemin and aptamer mixture was added 10 µL of 60 mm H_2_O_2_ to initiate the reaction. According to the following formula, the *K*_d_ values at different cases were obtained.
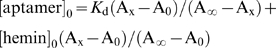



Where [aptamer]_0_ and [hemin]_0_ are the initial concentrations of aptamer and hemin; A_0_, A_∞_ and A_x_ are the absorbance (at 421 nm) for analyzing uncomplexed hemin (in the absence of aptamers), fully bound hemin (in the presence of extremely excess aptamers), and hemin bound partially by aptamers (in the presence of appropriate concentrations of aptamers).
